# A Systematic Review of Indications and Clinical Outcomes of Electrochemotherapy in Pancreatic Ductal Adenocarcinoma

**DOI:** 10.3390/cancers17030408

**Published:** 2025-01-26

**Authors:** Gianluca Rompianesi, Giuseppe Loiaco, Luigi Rescigno, Gianluca Benassai, Mariano Cesare Giglio, Silvia Campanile, Marcello Caggiano, Roberto Montalti, Roberto Ivan Troisi

**Affiliations:** 1 Department of Clinical Medicine and Surgery, Division of Minimally Invasive and Robotic HPB S and Gery, Transplantation Service, Federico II University Hospital, Via Sergio Pansini 5, 80131 Naples, Italy; gianluca.rompianesi@unina.it (G.R.); giuseppe.loiaco@unina.it (G.L.); luigi.rescigno@unina.it (L.R.); gianluca.benassai@unina.it (G.B.); mariano.giglio@unina.it (M.C.G.); silvia.campanile@unina.it (S.C.); marcello.caggiano@unina.it (M.C.); roberto.montalti@unina.it (R.M.); 2 Department of Public Health, Federico II University, Via Sergio Pansini 5, 80131 Naples, Italy

**Keywords:** cancer, pancreatic duct adenocarcinoma, electrochemotherapy, systematic review

## Abstract

Background: Pancreatic ductal adenocarcinoma (PDAC) is one of the most difficult cancers to treat, with a dismal 5-year survival rate of only 8–10%. This challenging prognosis highlights the urgent need for innovative therapeutic approaches to improve outcomes for patients with PDAC. Electrochemotherapy (ECT), which enhances intracellular chemotherapeutic uptake via electric pulses, has been explored for resectable, borderline resectable (BR), locally advanced (LA), recurrent, and metastatic PDAC, either as a complement to conventional treatments or as an alternative when these are not feasible or effective, offering possible benefits in symptomatic palliation and local tumor control. Methods: A systematic review was performed in accordance with PRISMA guidelines for studies assessing the efficacy of ECT in PDAC. After searching Embase, PubMed/MEDLINE, Scopus, and Web of Science, five studies with a combined total of 43 patients in various disease stages were identified. Results: ECT showed promise in improving tumor control, alleviating cancer-related pain, and improving quality of life. One study noted a trend towards tumor size reduction of 8.3% at one-month and 16.1% at six-months follow-up (*p* = 0.211 and *p* = 0.315), although these findings were derived from studies conducted without specific comparative control groups. Severity of complication was mainly mild (Clavien–Dindo I-II), while severe complications occurred in only 2.3% of patients. Median overall survival was reported in two studies as 8 months (range 2–19) and 11.5 months (range 1–74). ECT showed efficacy for symptom management, with 60% of patients reporting reduced pain/discomfort and 40% showing enhanced quality of life in one study, while another reported pain scores as decreasing from 6 to 3 at one month and to 2 at six months. Conclusions: ECT appears to be a new promising and safe adjunct treatment modality in PDAC management across different disease stages, with potential benefits in tumor control, cancer-related pain reduction, and quality of life. Further studies are warranted to validate these findings and identify patients who could benefit most.

## Simple Summary

Pancreatic ductal adenocarcinoma (PDAC) is among the most challenging malignancies to cure, with limited treatment options and poor prognosis. Electrochemotherapy (ECT) is a new treatment modality that combines the administration of chemotherapeutic drugs with electrical pulses delivered via needle electrodes, which penetrate the tumor to enhance drug uptake and improve tumor control. This systematic review highlights the potential of ECT to enhance local tumor control, provide symptomatic relief, and possibly prolong survival in PDAC patients. While preliminary findings are promising, further research is essential to validate these outcomes and optimize its clinical application.

## 1. Introduction

Pancreatic ductal adenocarcinoma (PDAC) continues to be one of the most challenging malignancies to treat, with a 5-year survival of merely 8–10% and dismal prognosis [1]. At diagnosis, most patients present with metastatic or locally advanced disease which precludes them from curative surgery; only 15% to 20% of cases are candidates for surgical resection [2].

Even with the most recent multi-agent chemotherapeutic regimens, including those combining radiotherapy, the overall survival of patients affected by advanced PDAC only ranges between 11 and 24 months, ultimately allowing a surgical resection in less than a third of patients [3].

Operable patients do significantly better, with 5-year survival rates of up to 20–25% after multimodal treatment [4].

In this scenario, there is an unmet need for innovative therapies that can provide local tumor control and palliation, while prolonging survival in a disease with historically low response rates to conventional systemic therapies. Current treatment guidelines for PDAC recommend a multimodal approach tailored to disease stage: surgical resection followed by adjuvant chemotherapy for resectable cases, neoadjuvant therapy for borderline resectable cases, and systemic chemotherapy regimens, such as FOLFIRINOX or Gemcitabine/Nab-Paclitaxel, for locally advanced or metastatic cases [5]. Despite these advancements, the prognosis remains poor, underscoring the necessity of exploring new therapeutic strategies [6].

Electrochemotherapy (ECT) represents a new therapeutic modality combining chemotherapy with the application of locally delivered electrical pulses. This strategy has been investigated as a therapeutic option in multiple neoplasms, including deep-located abdominal tumors [7].

ECT works by applying a series of needle electrodes into the tumor mass, which release high-voltage electric pulses capable of creating transient micropores in the cancer cell membranes through a process known as reversible electroporation; by briefly increasing membrane permeabilization, this allows intracellular concentrations of cytotoxic agents including Bleomycin or Cisplatin, increasing their local effects, while limiting systemic toxicity [8]. This can be applied by two routes: percutaneous, for superficial or accessible tumors; or intraoperatively, for deep-seated cancers, possibly under intraoperative imaging guidance [9].

Another local ablative technique, irreversible electroporation (IRE), uses similar principles of electrical pulses but without the concurrent administration of chemotherapy. IRE has been explored in locally advanced pancreatic cancer (LAPC) as a tool to induce cell death through the permanent disruption of cancer cell membranes, with studies reporting promising outcomes in terms of local tumor control and survival [10]. Although both ECT and IRE rely on electroporation technology, they differ significantly in mechanism and clinical application, with IRE being primarily ablative and ECT enhancing chemotherapeutic efficacy [11].

While ECT has demonstrated efficacy in a range of solid tumors, its clinical use in PDAC is still largely experimental [12,13]. In patients with LAPC, preliminary studies have demonstrated improvements in local disease control [14]. Although encouraging, these initial findings need to be more extensively validated to clarify the potential role of ECT in the treatment of PDAC.

The objective of this systematic review is to summarize the evidence regarding the indications, safety, and efficacy of ECT in PDAC across different disease stages, trying to define its role in this complex disease and highlight important areas for future research.

## 2. Methods

This systematic review was conducted according to the Preferred Reporting Items for Systematic Reviews and Meta-Analyses (PRISMA) 2020 guidelines [15]. The checklist is provided in Table S1 of the supplementary materials for reference.

The review protocol was prospectively registered on the PROSPERO database (identification number: CRD42024594419) [16].

A search across PubMed/MEDLINE, Scopus, Embase, and Web of Science was performed from January 2000 up to 23 October 2024. Unpublished data from ongoing trials were also sought through searches of trial registries, specifically ClinicalTrials.gov, the EU Clinical Trials Register (EU-CTR), and WHO International Clinical Trials Registry Platform (WHO-ICTRP). The search only included English-written publications and focused only on human clinical cases. We performed a search strategy with combinations of the terms “pancrea*” and “electrochemotherapy” or “ECT”, designed to cover the widest range of studies on ECT for PDAC. Additionally, we manually checked the reference lists of relevant studies to find more eligible research articles.

This systematic review examined the use of ECT in adult patients (≥18 years) of either gender and any ethnicity, with a histologically proven diagnosis of PDAC, regardless of disease stage. Eligible studies recorded resectable, borderline resectable (BR), and LAPC, as well as recurrent and metastatic PDAC cases. All types of study designs, comprising case series and case reports, were included in the search to ensure the broadest collection of evidence from the literature. Conference abstracts containing unpublished data were also included. Inclusion and exclusion criteria are illustrated in Table 1.

Two reviewers (GL, LR) independently applied eligibility criteria and resolved ambiguities through a third reviewer (GR) to enhance the reliability of study selection.

The information collected regarded study ID, first author, year of publication, design of the study, total number of patients, demographic data (gender, age, body mass index (BMI), ECOG performance status), and clinical and tumor characteristics (histological type, location and size of the tumor, vascular involvement), as well as disease stage and treatment indication, in order to provide a baseline characterization of the cohorts from the included studies.

Technical data regarding the applied ECT protocols included systemic treatments prior to ECT, procedural method and electrode arrangement, duration of ECT procedure, and type and total dose of chemotherapeutics agents. Postoperative and follow-up data included fundamental clinical information, such as hospital stay, complications, tumor response by size, and overall survival (OS), as well as quality of life following ECT therapy.

Pain and quality of life were evaluated in individual studies using specific scales, such as the DVPR scale and the EQ-5D-5L scale.

Two reviewers (GL and LR) performed data extraction using a predefined extraction format to ensure consistency, and any disagreements were resolved by consulting a third reviewer (GR).

To allow for the most appropriate assessment of each study type, we assessed the quality using tools consistent with the study design. The Newcastle–Ottawa Scale (NOS) was used to assess the quality of observational studies [17], while for non-randomized interventional and pilot studies, we used the MINORS criteria [18], and for conference abstracts, we assessed their quality with SANRA criteria [19]. To improve reliability, each study was independently assessed by two reviewers (GL and LR).

Given the heterogeneity among study designs, interventions, and outcomes from the included articles, a narrative synthesis—rather than a formal meta-analysis—was employed to interpret and summarize the findings. Results were reported as the median and—when feasible—interquartile range (IQR) to account for potential outliers and small sample sizes. Average ± standard deviation was used for data that demonstrated a normal distribution, with normality assessed using both Shapiro–Wilk and Anderson–Darling tests.

## 3. Results

Overall, 421 records were identified through the search strategy (413 in electronic databases—Embase (*n* = 94), PubMed/MEDLINE (*n* = 60), Scopus (*n* = 158), and Web of Science (*n* = 101). An additional eight records were identified from clinical trial registries, including ClinicalTrials.gov (*n* = 4), the EU Clinical Trials Register (*n* = 3), and the WHO International Clinical Trials Registry Platform (WHO-ICTRP, *n* = 1).

After excluding 171 duplicates and five articles published in languages other than English, as well as one withdrawn trial, we retained a set of records composed of 244 unique references.

Based on the predefined exclusion criteria, for the search for titles/abstracts and clinical trial reports, 235 out of 244 references were excluded. Specifically, 73 articles did not address PDAC, 57 were not primary research articles (e.g., commentaries, editorials, reviews, systematic reviews, or meta-analyses), 66 did not focus on ECT, and 39 were based on studies involving cell lines or animal models rather than human patients. No further relevant records were identified from manual reference searches.

**Figure 1:**
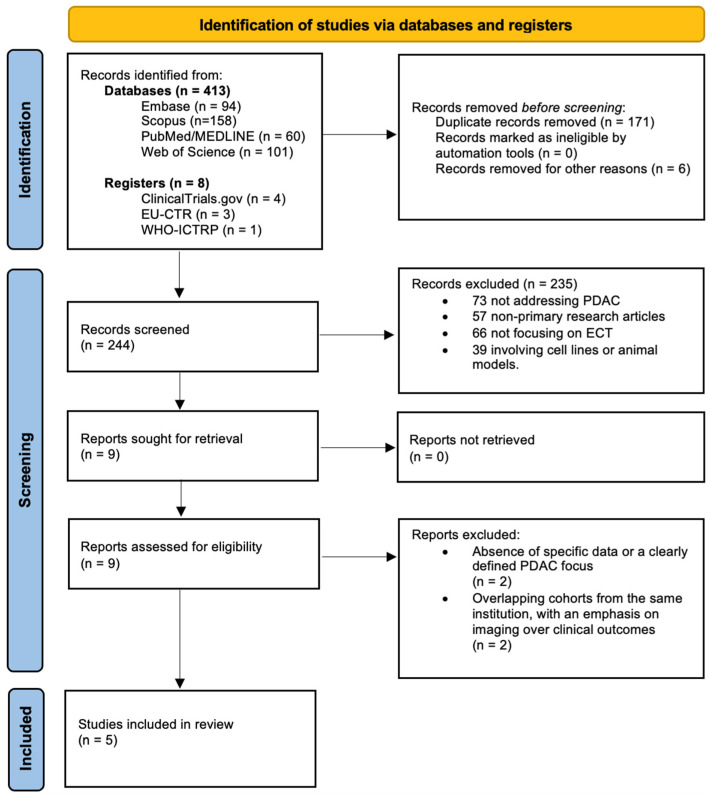
PRISMA flowchart for selecting and screening the resultant papers of the initial literature search.

Of the remaining nine reports, full-text analysis ruled out a further four studies either due to insufficient data being available (*n* = 2) [20,21] or due to overlaps in populations with other included studies, which primarily focused on imaging rather than clinical outcomes (*n* = 2) [14,22]. Finally, five studies satisfied all criteria for inclusion and were therefore part of the systematic review [23,24,25,26,27] (Figure 1).

### 3.1. Quality Assessment and Publication Bias

Among the reviewed studies, four provided moderate to good evidence on the feasibility, safety, and efficacy of ECT in PDAC, including pilot trials, phase I/II studies, and observational cohorts. Although the objectives were clear and outcomes consistent, methodologic limitations—including single-arm designs, lack of control groups or randomized clinical trials, and variable follow-up periods—affected the overall robustness of the evidence. One conference abstract provided only preliminary insights and was limited in terms of methodological rigor. There were no disagreements among reviewers in the quality assessment process (Table 2).

### 3.2. Study Characteristics

Each of the five studies that were included in this systematic review provided insights into ECT as a treatment for pancreatic cancer at different disease stages comprising resectable, BR, LAPC, recurrent, and metastatic PDAC (Table 3).

Izzo et al. [23] focused on surgical outcomes and local disease control in LAPC patients receiving intraoperative ECT after preoperative chemotherapy, evaluating its use to improve local disease control and overall survival (OS); Cebron et al. [24] evaluated ECT as an intraoperative adjunct to pancreaticoduodenectomy (PD) in resectable PDAC without vascular involvement, targeting high-risk resection margins with the intention of lowering local recurrence rates; Rudno-Rudzińska et al. [25] explored ECT (as well as other local ablative techniques) use throughout various disease stages, including BR-PDAC, LAPC, and recurrent PDAC, all treated with systemic chemotherapy prior to ECT; Casadei et al. [26] assessed quality of life outcomes including pain relief, and OS in ECT-treated LAPC patients following chemoradiotherapy.

Giardino et al. observed the effects of ECT on metastatic PDAC lesions at the abdominal wall in two cases previously treated with radiofrequency ablation (stage III pancreatic head carcinoma) and distal pancreatectomy with splenectomy (stage II pancreatic tail carcinoma) [25].

### 3.3. Patient Demographics

Across the five included studies, a total of 43 patients underwent ECT for pancreatic PDAC, with 46.5% being male. The average BMI was 23.2 Kg/m^2^ (±3.1 Kg/m^2^), although these data were inconsistently reported among studies. Performance status (when recorded) was ECOG 0 or 1, implying only mild physiologic restrictions.

### 3.4. Tumor Characteristics

According to the National Comprehensive Cancer Network (NCCN) classification [28], of the 43 cases examined, there were seven (16%) resectable PDAC; 1 BR-PDAC (2%); 31 LAPC (72%). The remaining patient were two cases (5%) of recurrent and two (5%) cases of metastatic disease.

Across the recruited studies, tumor size assessment was performed using differing methods of imaging (CT, MRI, and US). The median tumor size for all cases was 38.5 mm (IQR: 28.5–51.8 mm; Δ = 23.3 mm). The median tumor size for LAPC cases was 45.5 mm (IQR: 33.5–53.8 mm; Δ = 20.3 mm). Tumors in resectable and BR stages treated with surgical resection followed by ECT at high-risk margins had a lower median size of 24.0 mm (IQR: 20.0–28.3 mm; Δ = 8.3 mm), reflecting earlier-stage disease. The two local recurrent tumors were 30 and 40 mm in diameter, and the metastatic lesions were measured at 20 mm and 32 mm.

Concerning the site of the tumor, 59% of tumors were located in the pancreatic head, 31% in the body/tail, 5% in the uncinate process, and 5% (metastatic lesions) in the abdominal wall.

Vascular involvement was reported in all of the included studies, comprising 30 patients (69.8%), with all of them presenting with LAPC involving venous and/or arterial structures in [23,26]. Venous involvement (superior mesenteric vein and portal vein) was identified in 25 cases (83.3%), while arterial disease extension (celiac trunk and/or superior mesenteric artery) was found in 19 cases (63.3%).

### 3.5. ECT Protocols in PDAC Treatment

Out of the 43 patients, 34 (79.1%) were reported to have received a systemic pre-ECT treatment, which included chemotherapy or chemoradiotherapy [23,25,26]. Among them, 31 patients (91.2%) were staged as LAPC, 1 patient (2.9%) was staged as BR-PDAC, and 2 patients (5.9%) had local recurrent disease.

Chemotherapy regimens involved GEMOX in 17 (50.0%), FOLFIRINOX in 13 (38.2%), Gemcitabine/Nab-Paclitaxel in 2 (5.9%), Gemcitabine in 1 (2.9%), and FOLFOX in 1 patient (2.9%). One study also incorporated radiotherapy, delivering 54 Gy in 27 fractions to further improve outcomes [26].

The included studies demonstrated comparable methodological approaches in the application of ECT; nevertheless, certain methodological variations were also observed (Table 4).

ECT was performed under general anesthesia for open surgery procedures and under sedation for percutaneous approaches.

Intravenous infusions of chemotherapeutic agents used included Bleomycin (standardized to body surface area at 15,000 IU/m^2^) in 90.7% of patients and Cisplatin in the remaining 9.3%.

Electrode needles were placed directly into the tumor mass, either in a fixed or tailored geometric configuration.

All procedures incorporated ECG synchronization to ensure cardiac safety by aligning electric pulse delivery with the refractory period of the cardiac cycle, thus avoiding arrhythmia.

Izzo et al. [23] applied ECT by open laparotomy, with a midline incision for staging of disease and mobilization of pancreatic tumor. Intravenous bleomycin (15,000 IU/m^2^) was given according to the ESOPE protocol [29,30]. Various electrode types were employed, including fixed configurations such as linear or hexagonal, as well as variable geometries. Electrode placement was optimized through the use of dedicated planning software. Electroporation was carried out with 8–96 pulses of 100 µs each at 400–1000 V (910–1000 V/cm), applied at a repetition frequency of 1–5000 Hz. The procedure was completed within 8 to 40 min post-bleomycin administration.

Cebron et al. [24] administered ECT intra-operatively to the pancreatic posterior resection surface following PD. Intravenous Bleomycin (15 mg/m^2^) was the chemotherapeutic of choice. The resection area was covered with plate electrodes (30 mm in length, 8 mm in spacing) without injuring blood vessels. Electroporation application consisted of eight trains of 100 µs pulses applied at 960 V and 5000 Hz. Electrode placement was guided anatomically to landmarks adjacent to the resection sites.

Rudno-Rudzińska et al. [25] conducted ECT in an open-access manner under general plus epidural anesthesia, with intraoperative ultrasound (IOUS) to guide tumor mass location and electrode placement. Cisplatin, administered intravenously, served as the chemotherapeutic agent. The average procedure time of about 3 h reported in the reviewed cases included the entire process from laparotomy to completion.

Casadei et al. [26] performed ECT following laparotomy and IOUS to confirm tumor unresectability (NCCN criteria [28]). Needle electrode placement was guided by IOUS, after which 15,000 IU/m^2^ of Bleomycin was injected intravenously. Long needle electrodes (20 or 16 cm, active part 3–4 cm) were arranged in variable geometry. Electroporation was accomplished with a single train of eight pulses (100 µs, 1000 V/cm). The procedure was concluded within 8 to 40 min after the infusion of Bleomycin.

Giardino et al. [27] described two cases of percutaneous ECT for PDAC metastases to the abdominal wall. In both cases, intravenous Bleomycin was administered at a dosage of 15,000 U/m^2^, followed by five and eight electroporation applications, respectively. Further procedural details were not explicitly reported.

In three of the included studies, patients underwent additional chemotherapy following ECT, although details were not homogenously reported [23,25,26].

### 3.6. Tumor Response and Local Disease Control

Among the five studies in this systematic review, only Izzo et al. [23] provided detailed quantitative data on the dimensional response of pancreatic tumors treated with ECT. Tumor sizes were assessed at baseline (pre-ECT) and monitored throughout the follow-up period using CT and MRI at one- and six-months post-treatment. At baseline, the CT measurements showed an average tumor size of 50.6 mm, which then decreased to 46.4 mm (8.3% reduction) at one month and then to 42.5 mm (16.1% reduction) at six months. Although a trend towards tumor size reduction was noted, paired t-tests showed that these reductions were not statistically significant (*p* = 0.211 at month 1; *p* = 0.315 at month 6). MRI measurements reflected a similar pattern, with a baseline mean of 49.1 mm, decreasing to 47.6 mm (2.9% reduction) at one month and to 35.33 m (28.0% reduction) at six months. While the one-month MRI reduction was not statistically significant (*p* = 0.226), the six-month reduction reached significance (*p* = 0.009); nevertheless, this result was based on only five patients, limiting its reliability.

None of the included studies examined CA 19-9 levels pre- and post-ECT for biological response comparison.

Izzo’s study [23] also uniquely provided quantitative data on disease control outcomes evaluated using Response Evaluation Criteria in Solid Tumors (RECIST) 1.1 and Choi criteria [31,32]. At one-month post-treatment, 76% of patients had a partial response, while 20% achieved stable disease, and the remaining 4% could not complete the follow-up due to early mortality. By six-months follow-up, 44% of patients were in partial response, and 12% had stable disease, although none reached a complete response. The remaining 44% of patients did not survive until the second follow-up. Notably, at the six-month follow-up, the group of patients treated with variable geometry electrodes had better disease control, with 67% of patients exhibiting partial response or stable disease, compared to 46% in the fixed geometry electrode group. However, this difference did not reach statistical significance.

The other studies provided only very limited or descriptive measures of local control. For instance, in the study by Casadei et al. [26], four out of five (80%) patients had stable disease after ECT, and one progressed, but no further response evaluation points for the whole follow-up period were described. Similarly, Giardino et al.’s study offered only a qualitative assessment, noting a partial reduction in the size of the treated metastatic nodule in both cases at the 1-month follow-up [27].

### 3.7. Survival Outcomes

Two of the reviewed studies reported details regarding the OS from time of ECT treatment. Izzo et al. [23] reported a median OS of 11.5 months (range 1–74 months) without disclosing individual patient survival. In Casadei et al.’s cohort [26], the calculated OS median was 8 months (range 2–19 months). Although the remaining studies did not report OS specifically, some surrogate data were available. In the study by Cebron et al., patients receiving ECT targeting high-risk resection margins after PD were followed for at least 6 months after the intervention. At that time, three out of the seven (43%) patients experienced disease progression—two with liver metastasis and one with both liver metastasis and local recurrence—leading to two death events. Importantly, one 81-year-old patient was recurrence-free at 28 months after treatment, suggesting extended OS in the absence of progression. At the time of publication, five other patients were still alive [24].

Rudno-Rudzińska et al. reported a median OS of 26 months, calculated from the date of diagnosis rather than from the time of ECT, which complicates interpretation. Moreover, the diversity in disease stage and treatment modalities—from ECT alone, to ECT plus surgical resection, to ECT plus irreversible electroporation (IRE)—within this cohort limits conclusions regarding the specific impact of ECT on survival outcomes [25].

### 3.8. Postoperative Recovery

A pooled analysis of four out of five studies providing detailed postoperative data on ECT for PDAC [22,23,24,25], encompassing a total of 18 patients, revealed an overall complication rate of 50%. Notably, 44.4% of patients in this subgroup underwent ECT targeting high-risk resection margins after PD.

Across all five studies, 91.7% of observed complications were classified as minor to moderate according to Clavien–Dindo (grades I–II) [33] (Table 5). This included fever (32.6% of patients), delayed gastric emptying (18.6%), ascites (18.6%), pleural effusion (14%), and portal vein thrombosis (7%). The incidence of severe complications was as low as 2.3% (1 out 43 patients), represented by a grade B pancreatic fistula (according to the ISGPS classification [34]), classified as a Clavien–Dindo grade IIIa. It is noteworthy that both grade B pancreatic fistulas and portal vein thromboses were observed in patients who underwent ECT applied to high-risk resection margins following PD and without preoperative vascular tumor involvement.

Across the studies providing individual patient data, the combined median duration of postoperative stay following intraoperative ECT for pancreatic cancer was 9.5 days, with a range from 2 to 86 days [24,25,26,27]. Postoperative stay varied by disease stage and treatment approach. For resectable or BR-PDAC patients undergoing both surgical resection and intraoperative ECT targeting high-risk resection margins, the median postoperative stay was 10.0 days, with a range of 7 to 86 days [24,25].

Among patients with LAPC, the median stay was 7.0 days, ranging from 5 to 14 days, based on individual data from Casadei et al. and Rudno-Rudzińska et al. [25,26]. Furthermore, Izzo et al. reported a median stay of 11.7 days (range, 7–19 days) for LAPC cases treated with ECT, though individual patient data were not available for this cohort [23].

For recurrent disease, postoperative stays were reported at 7 and 12 days [25]. Finally, in metastatic cases treated with ECT, postoperative stays were as brief as 2 days [27].

### 3.9. Pain and Quality of Life After ECT in PDAC Treatment

Two studies reported on pain and quality of life in PDAC patients treated with ECT. In one study, median pain scores, measured by the DVPR scale [35], notably decreased from score 6 pre-ECT to 3 at one-month post-ECT and to 2 at six months, indicating substantial pain relief [23]. The second study, using the EQ-5D-5L scale [36], showed improvements in pain/discomfort in 3 out of 5 (60%) patients and an overall quality of life improvement in 2 out of 5 (40%) of patients post-ECT [26].

## 4. Discussion

This systematic review suggests that ECT could represent a potential complementary therapy for PDAC, a malignancy historically associated with poor treatment options and prognosis. ECT combines local electrical pulses and cytotoxic drugs to improve drug uptake within tumor cells, which may help overcome tumor resistance to systemic cancer treatments by increasing cell membrane permeability.

The studies summarized in this review demonstrate the broad applicability of ECT to different presentations of PDAC, whether resectable, BR, LAPC, recurrent, or metastatic. The adaptability of ECT to various stages of disease, as well as different clinical contexts, indicate that ECT may become a part of the arsenal of multimodal treatment algorithms for PDAC. However, despite the promising initial results regarding tumor response and patient outcomes, ECT in PDAC is still experimental and therefore more well-designed studies are warranted to confirm these findings and define the role of ECT.

The results suggested a beneficial effect of ECT upon tumor responsiveness and local disease control but were inconsistent between studies. For instance, Izzo et al. documented a reduction in tumor size at six months post-ECT, suggesting a moderate degree of local disease control [23]. However, this was the only study to report quantitative, statistically analyzed data, and the small sample size and short follow-up duration limited the generalizability of these findings. Most studies only reported qualitative data with descriptions of stable disease or partial responses, limiting standardization around metrics to assess response. Such variability complicated comparisons across the included studies and underscored the need for future research to employ uniform response criteria, such as the RECIST or Choi classification, as well as biomarkers, to improve consistency and accuracy in assessing ECT efficacy. Indeed, standardizing outcome measures could facilitate future meta-analyses and ultimately provide more definitive conclusions on ECT’s clinical benefits. None of the included studies reported data regarding post-treatment CA 19-9 serum levels, not allowing a thorough evaluation of the biological tumor response following ECT, which would be of great interest, especially in case of locally advanced PDAC.

Data on survival in patients treated with ECT remain limited and challenging to interpret. OS metrics were specified in only two studies, with median overall survivals of 11.5 months (range 1–74) and 8 months (range 2–19) after ECT [23,26]. This wide range indicates considerable heterogeneity in patient selection and treatment protocols among the studies.

Some ongoing randomized clinical trials are designed to evaluate the efficacy and safety of ECT in pancreatic cancer. A multicenter randomized trial in Italy is comparing laparoscopic ECT followed by systemic chemotherapy with standard chemotherapy alone for locally advanced pancreatic cancer, focusing on progression-free survival, quality of life, and tumor resectability [37]. Similarly, the IREC study in Poland is a randomized trial evaluating the safety and efficacy of ECT, calcium electroporation, and irreversible electroporation in non-resectable pancreatic cancer, with endpoints including progression-free survival, overall survival, and quality of life [38]. These trials are expected to provide robust evidence to define ECT’s role within multimodal treatment strategies.

Across studies, one of the most positive aspects of ECT is its safety profile. The vast majority of the observed complications were minor to moderate (Clavien–Dindo grades I–II), indicating that ECT is a low-risk intervention that may be safely incorporated into current PDAC treatment pathways. There was only one case of a grade IIIa complication (grade B pancreatic fistula), which developed in a patient undergoing ECT of high-risk resection margins after PD, a procedure known to be burdened by high surgical morbidity [24]. This case highlights the importance of carefully attributing complications, as they may not be directly related to ECT but rather to the complexity of the associated surgical procedures. Combined with the procedure’s brevity (8–40 min post-Bleomycin infusion), this low-risk and efficient profile underscores ECT’s feasibility as a possible addition to multimodal treatment strategies for PDAC. These findings are reassuring regarding the safety of ECT; however, the small sample size in each study and the limited long-term follow-up across studies prevented any definitive conclusions on the safety profile of ECT.

Another significant finding in this review is the variability in ECT protocols and pre-treatment regimens across studies, especially in the setting of advanced stages of disease. Prior to ECT, diverse chemotherapy regimens including GEMOX, FOLFIRINOX, and Gemcitabine/Nab-Paclitaxel, each known to modulate tumor response, were often administered. Although this approach reflects the intricacy of PDAC patient management in a real-world clinical setting, it complicates the ability to finely dissect the specific impacts of ECT on patients. Future studies with standardized pre-ECT chemotherapy protocols would be helpful in elucidating the contribution of ECT to local disease control and survival. Furthermore, three of the included studies [23,25,26] reported on patients receiving post-ECT chemotherapy, thereby further confounding the attribution of a recurrence or survival outcome to that observed after ECT alone.

Besides its role in local tumor control, ECT may also contribute in a palliative setting, alleviating pain and improving quality of life in many patients with advanced PDAC. Two of the reviewed studies indicated clinically considerable improvements in cancer-related pain and quality-of-life [23,26]. This is of clinical relevance, since controlling symptoms is a critical aspect of PDAC management, particularly in patients who are unfit for surgery and/or have exhausted other treatment options.

Overall, this systematic review introduces ECT as a viable but still investigational therapeutic tool that may ultimately enhance the management of PDAC through improved local control, prolonged survival, and better quality of life. The fact that 72% of patients across the reviewed studies were staged as LAPC underscores the potential role of ECT as a targeted approach in this challenging clinical context.

Considering the clinical experience gathered so far, ECT appears to hold significant potential in diverse settings and disease stages. It could become a useful adjunct to surgery in resectable patients in order to prevent local recurrence, be part of a multimodal neoadjuvant approach in combination with chemo-radiation and be performed in case of unresectable or LA-PDAC in order to achieve better local tumor control and enhance overall patient survival. However, this field of research is still in its early stages, with only five relevant studies identified in this systematic review, underscoring the need for further investigation to fully define its role in clinical practice.

## 5. Conclusions

While preliminary, the current evidence denotes a considerable potential for ECT in PDAC management, despite significant heterogeneity in the study designs, patient selection, and treatment protocols. Clinical implementation of ECT will require standardization with respect to timing, dosage of chemotherapeutic agent, and systemic chemotherapy. Well-designed, randomized clinical trials with stringent inclusion and exclusion criteria and protocols will be crucial to clearly define the efficacy and safety of ECT, allowing for incorporation into the treatment of PDAC.

In conclusion, ECT is emerging as an adjunct therapy for PDAC, particularly in locally advanced disease. Although still under investigation, it appears to be effective in enhancing local disease control and alleviating symptoms. Current evidence regarding its impact on overall survival remains limited, and further studies are needed to clarify its role in comparison to standard chemo- or chemoradiation therapies. ECT can also be effectively combined with palliative chemotherapy, expanding its potential applications in the multimodal management of PDAC. Furthermore, ECT should be considered in carefully selected patients, as part of multimodal treatment strategies, and within the framework of approved experimental protocols. Ongoing clinical trials are expected to provide more robust and reassuring data, further clarifying its role in the treatment of PDAC.

## Figures and Tables

**Table 1:** Inclusion and exclusion criteria used during title and abstract screening.

Inclusion Criteria	Exclusion Criteria
All types of study designs (including case reports and conference abstract with unpublished data)	Non-primary research (narrative reviews, systematic reviews and meta-analysis)
Adult patients aged ≥18 years	Pre-clinical studies
Any gender and ethnicity	Studies without outcome or efficacy data
Histologically confirmed PDAC diagnosis	Patients aged <18 years
All PDAC disease stage (resectable, BR-PDAC, LAPC, recurrent and metastatic lesions)	Non-PDAC conditions or non-ECT therapeutic approaches for PDAC

**Table 2:** Study quality assessment.

Study	Design	Assessment Tool	Score	Quality Rating
Giardino et al. (2011) [27]	Conference abstract	SANRA	4/12	Low
Casadei et al. (2020) [26]	Observational	NOS	6/9	Moderate
Izzo et al. (2021) [23]	Phase I/II	MINORS	13/16	Good
Rudno-Rudzińska et al. (2021) [25]	Pilot study	MINORS	10/16	Moderate
Cebron et al. (2024) [24]	Pilot Study	MINORS	11/16	Moderate

**Table 3:** Characteristics of the included studies.

Study	Sample Size	n. of Patients Treated with ECT	Indication	Treatment	Primary Outcomes
Giardino et al. (2011) [27]	2	2	Abdominal wall PDAC metastases	ECT with Bleomycin	Reduction in metastatic nodule
Casadei et al. (2020) [26]	5	5	Stable LAPC post-chemotherapy, not suitable for surgery	ECT with Bleomycin	Quality of life, pain control
Izzo et al. (2021) [23]	25	25	Stable LAPC post-chemotherapy, not suitable for surgery	ECT with Bleomycin	Local disease control, survival
Rudno-Rudzińska et al. (2021) [25]	13	4	PDAC in various stages (BR, LA, recurrent)	ECT with Cisplatin, ECT + CaEP	Safety and efficacy assessment
Cebron et al. (2024) [24]	7	7	Treatment of high-risk resection margins in resectable PDAC	ECT with Bleomycin	Reduction in local recurrence

**Table 4:** Procedures and device details.

Study	Procedure	Drug and Dose	Electrodes	Electric Pulse Parameters	Duration
Giardino et al. (2011) [27]	ECT of abdominal wall metastatic lesions through percutaneous approach.	Bleomycin 15,000 U/m^2^ intravenously (24 mg and 22 mg in two cases).	Not detailed.	Not detailed.	Not detailed
Casadei et al. (2020) [26]	ECT of LAPC through open surgery approach.	Bleomycin 15,000 IU/m^2^ intravenously	Electrodes system used: IGEA Cliniporator™ (long single needle electrodes 20 or 16 cm, 3–4 cm active part). IOUS used for planning electrodes configuration.	Eight pulses of 100 µs duration, 1000 V/cm, ECG-synchronized, impedance-adjusted.	8–40 min post- Bleomycin.
Izzo et al. (2021) [23]	ECT of LAPC through open surgery approach.	Bleomycin 15,000 IU/m^2^ intravenously.	Electrodes system used: IGEA Cliniporator™. Pre-planned electrodes configuration (fixed or variable geometry) with IGEA PULSAR software.	8–96 pulses of 100 µs duration, 400–1000 V, 1–5000 Hz, ECG synchronized.	8–40 min post- Bleomycin.
Rudno-Rudzińska et al. (2021) [25]	Open-access technique	Cisplatin, dose adjusted to patient-specific surface area.	Electrodes system used: ANGIODYNAMICS NanoKnife™ system. Specific configurations not detailed.	Not fully detailed; synchronization with P-wave in ECG reported.	Average operation time: 3 h.
Cebron et al. (2024) [24]	Intraoperative ECT targeting the posterior resection surface following PD.	Bleomycin 15 mg/m^2^ intravenously.	Electrodes system used: IGEA Cliniporator Vitae™ (plate electrodes 30 mm length, 8 mm spacing).	Eight pulses of 100 µs duration, 960 V, 5 kHz, ECG-synchronized.	Not detailed

**Table 5:** Complications following ECT alone or in combination with surgery.

Complication	Frequency	Clavien–Dindo
Pancreatic fistula ^1^	4.7% (2/43)	II–IIIa
Portal vein thrombosis ^2^	7.0% (3/43)	II
Deep vein thrombosis	2.3% (1/43)	II
Atrial fibrillation	2.3% (1/43)	II
Fever	32.6% (14/43)	I
Delayed gastric emptying	18.6% (8/43)	I
Ascites	18.6% (8/43)	I
Pleural effusion	14.0% (6/43)	I
Splenic infarction	7.0% (3/43)	I
Surgical site infection	4.7% (2/43)	I
Subcutaneous edema ^3^	4.7% (2/43)	I

^1^ Pancreatic fistula grade IIIa observed in Cebron’s et al. cohort (PD + ECT). ^2^ In 1 case out of 3 a partial PV thrombosis was observed. ^3^ ECT applied percutaneously.

## Data Availability

The data from this study are available from the Embase, PubMed/MEDLINE, Scopus, and Web of Science databases.

## Supplementary Materials

The following supporting information can be downloaded at: https://www.mdpi.com/article/10.3390/cancers17030408/s1, Table S1. PRISMA 2020 check list.

## References

[1] Siegel R.L., Miller K.D., Jemal A. (2020). Cancer Statistics, 2020. CA Cancer J. Clin..

[2] Mizrahi J.D., Surana R., Valle J.W., Shroff R.T. (2020). Pancreatic Cancer. Lancet.

[3] Stoop T.F., Theijse R.T., Seelen L.W.F., Groot Koerkamp B., van Eijck C.H.J., Wolfgang C.L., van Tienhoven G., van Santvoort H.C., Molenaar I.Q., Wilmink J.W. (2024). Preoperative Chemotherapy, Radiotherapy and Surgical Decision-Making in Patients with Borderline Resectable and Locally Advanced Pancreatic Cancer. Nat. Rev. Gastroenterol. Hepatol..

[4] Neoptolemos J.P., Kleeff J., Michl P., Costello E., Greenhalf W., Palmer D.H. (2018). Therapeutic Developments in Pancreatic Cancer: Current and Future Perspectives. Nat. Rev. Gastroenterol. Hepatol..

[5] Conroy T., Pfeiffer P., Vilgrain V., Lamarca A., Seufferlein T., O’Reilly E.M., Hackert T., Golan T., Prager G., Haustermans K. (2023). Pancreatic Cancer: ESMO Clinical Practice Guideline for Diagnosis, Treatment and Follow-Up. Ann. Oncol..

[6] Schepis T., De Lucia S.S., Pellegrino A., del Gaudio A., Maresca R., Coppola G., Chiappetta M.F., Gasbarrini A., Franceschi F., Candelli M. (2023). State-of-the-Art and Upcoming Innovations in Pancreatic Cancer Care: A Step Forward to Precision Medicine. Cancers.

[7] Bonferoni M.C., Rassu G., Gavini E., Sorrenti M., Catenacci L., Torre M.L., Perteghella S., Ansaloni L., Maestri M., Giunchedi P. (2021). Electrochemotherapy of Deep-Seated Tumors: State of Art and Perspectives as Possible “EPR Effect Enhancer” to Improve Cancer Nanomedicine Efficacy. Cancers.

[8] Mir L.M., Orlowski S. (1999). Mechanisms of Electrochemotherapy. Adv. Drug Deliv. Rev..

[9] Granata V., Fusco R., D’Alessio V., Simonetti I., Grassi F., Silvestro L., Palaia R., Belli A., Patrone R., Piccirillo M. (2023). Percutanous Electrochemotherapy (ECT) in Primary and Secondary Liver Malignancies: A Systematic Review. Diagnostics.

[10] Rai Z.L., Feakins R., Pallett L.J., Manas D., Davidson B.R. (2021). Irreversible Electroporation (IRE) in Locally Advanced Pancreatic Cancer: A Review of Current Clinical Outcomes, Mechanism of Action and Opportunities for Synergistic Therapy. J. Clin. Med..

[11] Tasu J.P., Tougeron D., Rols M.P. (2022). Irreversible Electroporation and Electrochemotherapy in Oncology: State of the Art. Diagn. Interv. Imaging.

[12] Granata V., Fusco R., Piccirillo M., Palaia R., Lastoria S., Petrillo A., Izzo F. (2014). Feasibility and Safety of Intraoperative Electrochemotherapy in Locally Advanced Pancreatic Tumor: A Preliminary Experience. Eur. J. Inflamm..

[13] Marty M., Sersa G., Garbay J.R., Gehl J., Collins C.G., Snoj M., Billard V., Geertsen P.F., Larkin J.O., Miklavcic D. (2006). Electrochemotherapy—An Easy, Highly Effective and Safe Treatment of Cutaneous and Subcutaneous Metastases: Results of ESOPE (European Standard Operating Procedures of Electrochemotherapy) Study. Eur. J. Cancer Suppl..

[14] Granata V., Fusco R., Setola S.V., Piccirillo M., Leongito M., Palaia R., Granata F., Lastoria S., Izzo F., Petrillo A. (2017). Early Radiological Assessment of Locally Advanced Pancreatic Cancer Treated with Electrochemotherapy. World J. Gastroenterol..

[15] Page M.J., McKenzie J.E., Bossuyt P.M., Boutron I., Hoffmann T.C., Mulrow C.D., Shamseer L., Tetzlaff J.M., Akl E.A., Brennan S.E. (2021). The PRISMA 2020 Statement: An Updated Guideline for Reporting Systematic Reviews. BMJ.

[16] Loiaco G. A Systematic Review of Indications and Clinical Outcomes of Electrochemotherapy in Pancreatic Duct Adenocarcinoma. PROSPERO 2024 CRD42024594419. https://www.Crd.York.Ac.Uk/Prospero/Display_record.Php?ID=CRD42024594419.

[17] Wells G.A., Shea B., O’Connell D., Peterson J., Welch V., Losos M., Tugwell P. The Newcastle-Ottawa Scale (NOS) for Assessing the Quality of Nonrandomised Studies in Meta-Analyses; Department of Epidemiology and Community Medicine, University of Ottawa, Ottawa, Canada; 2000. http://www.ohri.ca/programs/clinical_epidemiology/oxford.asp.

[18] Slim K., Nini E., Forestier D., Kwiatkowski F., Panis Y., Chipponi J. (2003). Methodological Index for Non-randomized Studies (*MINORS*): Development and Validation of a New Instrument. ANZ J. Surg..

[19] Baethge C., Goldbeck-Wood S., Mertens S. (2019). SANRA—A Scale for the Quality Assessment of Narrative Review Articles. Res. Integr. Peer Rev..

[20] Skarlatos I., Kyrgias G., Mosa E., Provatopoulou X., Spyrou M., Theodorou K., Lepouras A., Gounaris A., Koukourakis M. (2011). Electrochemotherapy in Cancer Patients: First Clinical Trial in Greece. In Vivo.

[21] Jocius D., Sileikis A., Poskus E., Strupas K. (2022). Electrochemotherapy (ECT) Use in Deep Seated Tumors. Cardiovasc. Interv. Radiol..

[22] Granata V., Fusco R., Setola S.V., Palaia R., Albino V., Piccirillo M., Grimm R., Petrillo A., Izzo F. (2019). Diffusion Kurtosis Imaging and Conventional Diffusion Weighted Imaging to Assess Electrochemotherapy Response in Locally Advanced Pancreatic Cancer. Radiol. Oncol..

[23] Izzo F., Granata V., Fusco R., D’Alessio V., Petrillo A., Lastoria S., Piccirillo M., Albino V., Belli A., Tafuto S. (2021). Clinical Phase I/II Study: Local Disease Control and Survival in Locally Advanced Pancreatic Cancer Treated with Electrochemotherapy. J. Clin. Med..

[24] Cebron Z., Djokic M., Petric M., Cemazar M., Bosnjak M., Sersa G., Trotovsek B. (2024). Intraoperative Electrochemotherapy of the Posterior Resection Surface after Pancreaticoduodenectomy: Preliminary Results of a Hybrid Approach Treatment of Pancreatic Cancer. Bioelectrochemistry.

[25] Rudno-Rudzińska J., Kielan W., Guziński M., Płochocki M., Antończyk A., Kulbacka J. (2021). New Therapeutic Strategy: Personalization of Pancreatic Cancer Treatment-Irreversible Electroporation (IRE), Electrochemotherapy (ECT) and Calcium Electroporation (CaEP)—A Pilot Preclinical Study. Surg. Oncol..

[26] Casadei R., Ricci C., Ingaldi C., Alberici L., Di Marco M., Guido A., Minni F., Serra C. (2020). Intraoperative Electrochemotherapy in Locally Advanced Pancreatic Cancer: Indications, Techniques and Results—A Single-Center Experience. Updates Surg..

[27] Giardino A., Frigerio I., Girelli R., Salvia R., Martini P.T., Barbi E., Bassi C. (2011). Abdominal Wall Metastasis from Pancreatic Carcinoma Treated with Electrochemotherapy (ECT): Report of Two Cases. HPB.

[28] National Comprehensive Cancer Network NCCN Guidelines for Pancreatic Adenocarcinoma. https://www.nccn.org/professionals/physician_gls/pdf/pancreatic.pdf.

[29] Mir L.M., Gehl J., Sersa G., Collins C.G., Garbay J.-R., Billard V., Geertsen P.F., Rudolf Z., O’Sullivan G.C., Marty M. (2006). Standard Operating Procedures of the Electrochemotherapy: Instructions for the Use of Bleomycin or Cisplatin Administered Either Systemically or Locally and Electric Pulses Delivered by the CliniporatorTM by Means of Invasive or Non-Invasive Electrodes. Eur. J. Cancer Suppl..

[30] Gehl J., Sersa G., Matthiessen L.W., Muir T., Soden D., Occhini A., Quaglino P., Curatolo P., Campana L.G., Kunte C. (2018). Updated Standard Operating Procedures for Electrochemotherapy of Cutaneous Tumours and Skin Metastases. Acta Oncol. (Madr).

[31] Eisenhauer E.A., Therasse P., Bogaerts J., Schwartz L.H., Sargent D., Ford R., Dancey J., Arbuck S., Gwyther S., Mooney M. (2009). New Response Evaluation Criteria in Solid Tumours: Revised RECIST Guideline (Version 1.1). Eur. J. Cancer.

[32] Choi H., Charnsangavej C., Faria S.C., Macapinlac H.A., Burgess M.A., Patel S.R., Chen L.L., Podoloff D.A., Benjamin R.S. (2007). Correlation of Computed Tomography and Positron Emission Tomography in Patients with Metastatic Gastrointestinal Stromal Tumor Treated at a Single Institution with Imatinib Mesylate: Proposal of New Computed Tomography Response Criteria. J. Clin. Oncol..

[33] Dindo D., Demartines N., Clavien P.-A. (2004). Classification of Surgical Complications. Ann. Surg..

[34] Bassi C., Marchegiani G., Dervenis C., Sarr M., Abu Hilal M., Adham M., Allen P., Andersson R., Asbun H.J., Besselink M.G. (2017). The 2016 Update of the International Study Group (ISGPS) Definition and Grading of Postoperative Pancreatic Fistula: 11 Years After. Surgery.

[35] Buckenmaier C.C., Galloway K.T., Polomano R.C., McDuffie M., Kwon N., Gallagher R.M. (2013). Preliminary Validation of the Defense and Veterans Pain Rating Scale (DVPRS) in a Military Population. Pain Med..

[36] Szende A., Janssen B., Cabases J. (2014). Self-Reported Population Health: An International Perspective Based on EQ-5D.

[37] Izzo F., Granata V., Fusco R., D’Alessio V., Petrillo A., Lastoria S., Piccirillo M., Albino V., Belli A., Nasti G. (2021). A Multicenter Randomized Controlled Prospective Study to Assess Efficacy of Laparoscopic Electrochemotherapy in the Treatment of Locally Advanced Pancreatic Cancer. J. Clin. Med..

[38] Rudno-Rudzińska J., Kielan W., Guziński M., Kulbacka J. (2021). Effects of Calcium Electroporation, Electrochemotherapy, and Irreversible Electroporation on Quality of Life and Progression-Free Survival in Patients with Pancreatic Cancer: IREC Clinical Study. Adv. Clin. Exp. Med..

